# DLPFC stimulation alters large-scale brain networks connectivity during a drug cue reactivity task: A tDCS-fMRI study

**DOI:** 10.3389/fnsys.2022.956315

**Published:** 2022-10-06

**Authors:** Ghazaleh Soleimani, Farzad Towhidkhah, Mohammad Ali Oghabian, Hamed Ekhtiari

**Affiliations:** ^1^Department of Biomedical Engineering, Amirkabir University of Technology, Tehran, Iran; ^2^Iranian National Center for Addiction Studies, Tehran University of Medical Science, Tehran, Iran; ^3^Neuroimaging and Analysis Group, Research Center for Molecular and Cellular Imaging, Tehran University of Medical Sciences, Tehran, Iran; ^4^Department of Psychiatry, University of Minnesota, Minneapolis, MN, United States; ^5^Laureate Institute for Brain Research, Tulsa, OK, United States

**Keywords:** transcranial direct current stimulation (tDCS), large-scale network, frontoparietal network (ECN), default mode network (DMN), ventral attention network (VAN), methamphetamine use disorder (MUD), drug cue reactivity

## Abstract

Transcranial direct current stimulation (tDCS) is a promising intervention for reducing craving/consumption in individuals with substance use disorders. However, its exact mechanism of action has not yet been well explored. We aimed to examine the network-based effects of tDCS while people with methamphetamine use disorders (MUDs) were exposed to drug cues. In a randomized, double-blind sham-controlled trial with a crossover design, 15 participants with MUDs were recruited to receive 20 min of active/sham tDCS with an anode/cathode over F4/F3. MRI data, including structural and task-based functional MRI during a standard drug cue-reactivity task, were collected immediately before and after stimulation sessions. Craving scores were also recorded before and after MRI scans. Individualized head models were generated to determine brain regions with strong electric fields (EFs). Using atlas-based parcellation of head models, averaged EFs were extracted from the main nodes of three large-scale networks that showed abnormalities in MUDs; executive control (ECN), default mode (DMN), and ventral attention (VAN) networks. Main nodes with high EF intensity were used as seed regions for task-based functional connectivity (FC) [using generalized psychophysiological interaction (gPPI)] and activity [using a general linear model (GLM)] calculations. Subjective craving showed a significant reduction in immediate craving after active (–15.42 ± 5.42) compared to sham (–1 ± 2.63). In seed-to-whole brain results, the PFC node in ECN showed an enhanced PPI connectivity with precuneus and visual cortex; the cluster center in MNI (6, –84, –12); the PFC node in DMN showed a decreased PPI connectivity with contralateral parietal cortex;(–48, –60, 46). ROI-to-ROI results showed increased PPI connectivity within/between ECN-VAN while connectivity between ECN-DMN decreased. In line with connectivity, functional activity in the right PFC node in DMN decreased after tDCS while activity in PFC nodes of ECN/VAN increased. EF calculations in PFC nodes revealed that EF in DMN was outward, while the direction of EFs was inward in ECN/VAN. This study provides new insight into neural circuitry underlying MUDs that can be modulated by tDCS at the network level and specifically suggests that bilateral tDCS increases cortical excitability in ECN and VAN, while it has opposite effects on DMN that may be related to the direction of EFs.

## Introduction

A majority of people with substance use disorders (SUDs) attempting traditional treatment options such as pharmacotherapy are at risk for relapse, even after long periods of abstinence, when they return to environments with drug-related cues (Fuchs et al., 2008). With respect to the lack of clinically reliable evidence for the effectiveness of commonly used interventions in SUDs, the identification of new treatments is needed. The neural substrate of cognitive dysfunctions, which are particularly pronounced during early periods of abstinence, is a core component of treatment options for drug addiction. Recent advancements in human neuroscience have provided novel therapeutic methods such as non-invasive brain stimulation (NIBS) techniques for targeting neural and cognitive processes involved in SUDs (Verdejo-Garcia et al., 2019).

Transcranial direct current stimulation (tDCS) is a widely used NIBS technology that delivers weak direct currents to the brain through the electrodes attached to the scalp (Nitsche and Paulus, 2000). It has been shown that tDCS can induce changes in neuronal excitability (Nitsche and Paulus, 2000) and ultimately modulate drug-related behaviors, such as self-reported craving or drug consumption (Chen et al., 2020; Kim and Kang, 2020). However, tDCS effects on the functional organization related to cue reactivity and their relevance for addiction symptoms are still preliminary. Integrating tDCS with functional magnetic resonance imaging (fMRI) [see Esmaeilpour et al. (2020) for more information] has provided new opportunities for optimally targeting drug-related functions.

One of the most commonly used paradigms for investigating brain functions underlying SUDs is the fMRI drug cue reactivity task (Ekhtiari et al., 2016). Changes in functional connectivity (FC) within or between large-scale networks in response to a change in the experimental condition (e.g., drug vs. neutral cues), called psychophysiological interaction (PPI), can help determine the task-related coupling between different parts of the brain (Friston et al., 1997). Although the effects of the drug cue reactivity task on FC have been investigated in previous drug-related studies such as cannabis (Filbey and Dunlop, 2014), nicotine (Janes et al., 2010; Bourque et al., 2013), cocaine (Kaag et al., 2018), and alcohol use disorders (Courtney et al., 2013; Bach et al., 2015), recent NIBS research has revealed that tDCS could modulate these neural substrates of drug cue reactivity (Yang et al., 2017).

Despite promising therapeutic results for tDCS in treatment of SUDs, there is no consensus regarding optimal electrode montages as an important factor that can affect stimulation outcomes (Shahbabaie et al., 2018b). However, considering our updated systematic review (Ekhtiari et al., 2019), by May 2022, 66 out of 75 trials (88%) that successfully stimulated drug-related behaviors such as drug craving or drug consumption used symmetric (bilateral DLPFC stimulation by placing electrodes over F3 and F4 locations, 79%) or asymmetric (unilateral DLPFC montages by placing the anode over F4/F3 and the cathode over the contralateral supraorbital area, 21%) electrode montages. Personalized computational head models showed that electrode location could affect current flow to the targeted areas, and the distribution of the current through the brain can affect stimulation outcomes (Mosayebi-Samani et al., 2021). For example, field strength was related to the response to bilateral tDCS over DLPFC such that higher electric field intensity correlated with greater BOLD signal change in the drug > neutral contrast in methamphetamine use disorders (MUDs) (Esmaeilpour et al., 2020).

Based on the computational head models (Datta et al., 2009), conventional tDCS produces diffuse brain current flow, and stimulation outcomes may be understood as modulation of global networks (Soleimani et al., 2021a). Previous fMRI studies have revealed that three networks—frontoparietal executive control network (ECN) for processing of exogenous stimuli, default mode network (DMN) involved in internally relevant stimuli as well as the self-monitoring process, and salience ventral attention network (VAN) implicated in attentional resource allocation between ECN and DMN—have received the most attention in SUDs (Reese et al., 2019; Bolton et al., 2020), which can be considered as stimulation targets in tDCS studies (Peña-Gómez et al., 2012; Kunze et al., 2016). One crucial mechanism underlying addiction is the coupling between the main nodes of these large-scale networks in response to drug-related cues, and applying stimulation over the DLPFC can modulate the interaction (activity/connectivity) between these network nodes (Peña-Gómez et al., 2012; Shahbabaie et al., 2018a; Abellaneda-Pérez et al., 2020). However, the role of large-scale brain networks in response to electrical stimulation and cue exposure is poorly understood.

In this study, the main goal was to examine the effects of tDCS on cue-induced craving and network-based FC between regions involved in the cognitive process related to cue exposure. We aimed to explore whether bifrontal tDCS (anode/cathode over F4/F3) modulates drug craving and FC within and between three main large-scale network nodes (ECN, DMN, and VAN) among a group of participants with MUDs based on the combination of fMRI drug cue reactivity data and tDCS in a randomized cross-over sham-controlled trial. Little information is available to guide the selection of the left or right hemisphere to place the anode as the stimulating electrode with excitatory effects. Both left and right-sided tDCS showed promising results for modulating addictive behaviors (Ekhtiari et al., 2019). Here, the right hemisphere was selected for placing the anode because (1) most of the previous tDCS studies in addiction medicine targeted the right DLPFC using a symmetric montage (anode/cathode over F4/F3) (Ekhtiari et al., 2019), and (2) in alcohol research, for example, there has been a unique emphasis on stimulating the right DLPFC (Klauss et al., 2014, 2018) with positive effects on reducing cue-reactivity (Wietschorke et al., 2016). Based on the previous findings, we hypothesized that active tDCS over DLPFC decreases drug craving compared to sham through increasing coupling within and between regions involved in task-positive networks, including ECN and VAN, and reducing connectivity within DMN as a task-negative large-scale network.

## Materials and methods

### Participants

Fifteen subjects with MUDs (all male; mean ± SD age: 31.33 ± 5.24 years) participated in the study, all of whom had a DSM-IV-TR (American Psychiatric Association, 2010) diagnosis of methamphetamine dependence and were under a course of abstinent-based therapy. The participants were recruited from the Omid Javeed residential addiction treatment center, Tehran Welfare Organization. This study was approved by the Ethics Committee of the Tehran University of Medical science, and all subjects gave their written consent to the experiment according to the Declaration of Helsinki. The trial was registered at the WHO registry for clinical trials (IRCT code: 2012102011172N1).

The inclusion criteria for this study were as follows: (1) To be male, (2) Persian-speaking, (3) diagnosed with MUD (last 12 months), (4) admitted to residential abstinence-based treatment for MUD and abstinence from methamphetamine for at least 1 week, and (5) willing and capable of interacting with the informed consent process. On the other hand, the exclusion criteria were as follows: (1) Any physical illness such as a brain tumor, active skin diseases, or scars near the electrode locations, (2) any current medication that may affect cognitive functioning, (3) unwillingness or inability to complete any of the major aspects of the study protocol, including drug cue rating, behavioral assessment, or magnetic resonance imaging (i.e., due to claustrophobia, or metal brain implants or pacemaker), (4) lifetime diagnosis with any psychotic disorder, and (5) self-reported abstinence from methamphetamine for more than 6 months.

### Design and procedure

**FIGURE 1:**
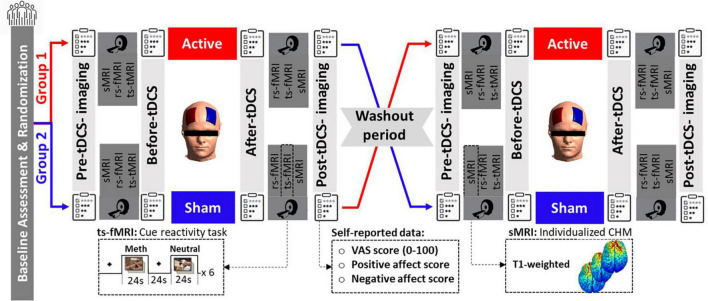
Data acquisition procedure. Fifteen participants with methamphetamine use disorder underwent real and sham tDCS randomly on separate days. tDCS was applied for 20 min at 2 mA through 5 × 7 cm^2^ sponge electrodes with the anode over F4 and the cathode over F3. In this clinical cross-over, double-blinded, sham control trial, neuroimaging data, including structural MRI (sMRI), resting-state (rs-fMRI), and task-based (ts-fMRI) fMRI data, were collected in a pre-stimulation/post-stimulation design. fMRI task was a standard cue-reactivity task with a pictorial block design that consisted of meth vs. neutral stimuli, and sMRI data were included in T1-weighted data used for creating individualized computational head models. Self-report data, including VAS and PANAS, were also collected before and after stimulation. sMRI, structural MRI; rs-fMRI, resting-state fMRI; ts-fMRI, task-based fMRI; CHM, computational head model; VAS, visual analog scale; PANAS, positive and negative affect score.

The data acquisition procedure is illustrated in Figure 1. This study used a randomized, double-blind, sham-controlled cross-over design. Clinical assessment and randomization were performed at the baseline after receiving each subject’s written consent letter. Participants received both active and sham bilateral DLPFC stimulation in two sessions on different days in the same daily context with at least a 1-week washout. The stimulation order was randomized and counterbalanced.

In each stimulation session, MRI data—structural (T1-weighted MRI, which was used for generating computational head models), resting-state, and task-based fMRI (a block design drug cue reactivity task)—were collected before and after tDCS (active or sham). Subjective craving for methamphetamine was assessed by the Visual Analog Scale (VAS, scored 0–100 where 0 indicated no craving and 100 represented extreme craving) at four-time points immediately before and after MRI scans; before first imaging (pre-tDCS imaging), before tDCS (after first imaging), after tDCS (before the second scan), and after second imaging (post-tDCS imaging). A positive and negative affect score (PANAS) was also collected at two different time points, before and after tDCS. Additionally, at the end of each session, tDCS side effects were also assessed for each individual. It is also worth mentioning that the full experimental protocol (as shown in Figure 1) included resting-state fMRI scans before the fMRI drug cue reactivity task and before and after active and sham tDCS sessions. Resting-state data are not included in the current manuscript but are reported elsewhere (Shahbabaie et al., 2018a).

### Transcranial direct current stimulation

Transcranial direct current stimulation was applied by a saline-soaked pair of surface sponge electrodes (5 × 7 cm^2^) and delivered by a battery-driven stimulator (ActivaDose II Iontophoresis Delivery Unit, USA). For bilateral DLPFC stimulation, the anode electrode was placed over F4, whereas the cathode electrode was placed over F3 according to the 10–20 standard system for EEG electrode placement, with the long axis of the pad pointing toward the vertex of the head. The electrodes were fixed to the scalp using multiple rubber bands. During active tDCS, a constant direct current of 2 mA was applied for 20 min, including a 30 s ramp up and down. The electrode placement was identical for sham stimulation; however, after a 30 s ramp up, the current was directly ramped down (yielding sensations typically elicited by tDCS). Then, the stimulator automatically switched off. A randomization sequence and code were generated by a research assistant in the data management team who did not participate in data collection to guarantee the double-blinding. Both participants and the person who administered the stimulation did not know which intervention (active or sham) was applied.

### Image acquisition

The experiment was performed with a 3.0 T MRI scanner (Siemens TIM Trio) at the Neuroimaging and Analysis Group at Tehran University of Medical Science. High resolution anatomical T1-weighted images were acquired with the following parameters: repetition time (TR) = 1,800 ms; echo time (TE) = 3.4 ms; field of view (FOV) = 256 × 256 mm^2^; flip angle (FA) = *7*; slice thickness = 1 mm; number of slices = 176. No head motion correction was performed for the aforementioned anatomical scans. Task-based fMRI data using a standard T2* weighted echo-planar imaging (EPI) sequence were acquired with the following parameters: TR = 3,000 ms, TE = 30 ms, 64 × 64 matrices, Flip Angle = 90, Field of view = 192 mm, in-plane resolution of 3 mm^2^ and 3 mm thickness. A total of 196 continuous EPI volumes were acquired for each fMRI session. In both MRI scans (before and after tDCS), task-based fMRI were collected immediately after resting-state fMRI (number of volumes = 200; number of slices = 40; TR/TE = 2,200/30 ms; percentage phase FOV = 100; matrix size = 64 × 64; slice thickness = 3 mm; interstice gap = 0 mm; FA = 90; spatial resolution = 3 × 3 × 3 mm^3^; FOV = 192 × 192 mm^2^).

### Structural magnetic resonance imaging analysis for generating computational head models

High-resolution T1-weighted MR images and SimNIBS 3.1 software were used for generating personalized computational head models (Thielscher et al., 2015). The meshes consisted of six main tissues: white matter (WM), gray matter (GM), cerebrospinal fluid (CSF), skull, vitreous bodies of the eyes, and skin using the “headreco” function in SPM 12 combined with the CAT12 toolbox. The anatomical accuracy of the final segmentations was visually controlled slice by slice against the high-resolution T1-weighted MR images. Segmented surfaces were used to create tetrahedral volume meshes. About 3 million tetrahedra meshes were assigned to each personalized head model. The rectangular 5 × 7 pads with a 1 mm thickness were modeled virtually and placed on top of the scalp of each realistic head model (anode/cathode over F4/F3) with the long axis of the pad pointing toward the vertex of the head. Based on previously reported values, the following isotropic conductivity values (in S/m) were used for the simulations: WM = 0.126, GM = 0.275, CSF = 1.654, skull = 0.010, skin = 0.465, eyeballs = 0.5 (Opitz et al., 2015).

To simulate electric field distribution patterns, the current strength of 2 mA was considered, and *EF* = −∇⁡φ was solved by applying the finite element solver (FEM) based on assuming a quasi-static regime (Opitz et al., 2015). To quantify electric field intensity, the tangential electric field (absolute value), as an indicator of electric field strength, and the radial electric field (normal component perpendicular to the cortical surface), which reflects currents either entering or leaving the cortex, were calculated for each individual. Finally, simulation results were converted into the standard average space, the average surface of FreeSurfer,^1^ to make head models comparable for further group-level analysis. The results were visualized by Gmsh (Geuzaine and Remacle, 2009) and MATLAB (version 2019b, The Math Works Inc.).

### Atlas-based parcellation of computational head models

With respect to the role of functional networks, including frontoparietal (ECN), DMN, and VAN in response to cognitive intervention such as tDCS, large-scale network parcellation was used for calculating electric field distribution patterns at the network level. Inspired by Soleimani et al. (2021a), we applied the Yeo7-2011 atlas to head models to determine the topology of the ECN, DMN, and VAN in the left and right hemispheres (Yeo et al., 2011). In the next step, Schaeffer-400-2018 was applied for a finer parcellation of the networks. Subregions placed adjacent to each other were merged to form the main nodes of the networks. Subsequently, the mean and standard deviation (SD) of the electric fields (EFs) were calculated within each main node across the population. Furthermore, main node masks in each large-scale network were converted to the NIFTI format. This was done by SPM 12 and saved for further functional analysis in the following steps.

### Drug cue-reactivity task paradigm

A block design paradigm was used for a drug cue reactivity task to evaluate tDCS effects on the neural response while participants watched two categories of stimuli: meth-related or neutral, in a pseudo-randomized order (Ekhtiari et al., 2010). In the scanner, participants viewed an alternating sequence of meth-related pictures (six blocks) and neutral images (six blocks) with 12 periods of rest separating the blocks from one another. During the rest period (a 24 s blank screen), a fixation cross was displayed at the screen center. Each meth or neutral block lasted for 24 s and consisted of four pictures of the same category (meth or neutral). To ensure participants were awake during the fMRI task, one of the pictures was marked in each block, and participants were asked to press a button as soon as they saw the mark. The whole task incorporated six meth-related and six neutral blocks with four pictures of each category in every block (i.e., 12 blocks in total) and took approximately 9.6 min.

### Magnetic resonance imaging preprocessing

Both structural and functional MRI data were analyzed with the CONN FC toolbox version 20.b (Whitfield-Gabrieli and Nieto-Castanon, 2012). The default CONN preprocessing pipeline was used based on employing functions from the SPM version 12 toolbox.^2^

For fMRI, preprocessing included (1) functional realignment and unwarping, (2) slice timing correction, (3) outlier detection, (4) segmentation and normalization to MNI space, and (5) smoothing. (1) In the realignment and unwarping step, all scans were co-registered to the first volume as a standard reference image. (2) To correct any temporal misalignment during the scanning session, slice timing correction was also applied. (3) The Artifact Detection Tools (ART) scrubbing procedure (implemented in CONN toolbox) was used to detect potential motion outliers in the data (including 12 motion parameters: x, y, z, pitch, roll, yaw, and their first order derivatives). Acquisitions with framewise displacement above 0.9 mm were flagged as outliers. If the number of volumes flagged as outliers divided by the total number of volumes was greater than 25%, that subject was excluded from the analysis. (4) Functional and structural data were normalized to MNI space and segmented into gray matter, white matter, and CSF using the mean BOLD signal as a reference image for functional data and T1-weighted MRI as a reference for structural data. After segmentation and normalization, all segmented tissues were checked visually one by one to detect incoherent deformation. (5) Finally, functional smoothing was performed with an 8 mm full width half maximum (FWHM) Gaussian kernel.

After preprocessing, the denoising procedure combined two general steps: (1) Linear regression of potential confounding effects in the BOLD signal (the CompCor method, which includes noise components from cerebral white matter and cerebrospinal areas, estimated subject-motion parameters, and outlier scans or scrubbing procedure) and (2) temporal band-pass filtering with a 0.01–0.1 Hz filter to focus on slow frequency fluctuations while minimizing the influence of physiological, scanner drift, head motion, and other noise sources.

For preprocessing of the structural images, anatomical images were centered, segmented, and normalized to the standard MNI space. After segmentation and normalization, normalized images were checked one by one to detect incoherent deformation.

### Task-based functional connectivity analysis

To investigate functional connectivity during the cue reactivity task, a seed-to-whole brain (seed-to-voxel) and region of interest to regions of interest (ROI-to-ROI) approaches were employed for generalized psychophysiological interaction (gPPI) in the CONN toolbox (Whitfield-Gabrieli and Nieto-Castanon, 2012). Task conditions were modeled by boxcar functions of the cue reactivity task convolved with a canonical hemodynamic response function (HRF). At the subject level, on the meth > neutral contrast, a weighted General Linear Model (GLM) was used for each subject to measure multivariate regression between the seed and each voxel or predefined ROI (as a source and a target, respectively) in a given context (here, the drug cue reactivity task). The seed region’s time course (as a physiological term), task time course (as a psychological term), and the interaction between task and BOLD signal in the seed region (as a psychophysiological (PPI) term) were considered in the gPPI design matrix. The BOLD signal of white matter and CSF and motion parameters were used as covariates to remove unwanted motion and physiologic artifact effects. For the second-level analysis, time (pre- and post-tDCS) by intervention (active vs. sham) interaction was calculated. At the voxel-level, *P* uncorrected < 0.001, and cluster-size false discovery rate (FDR) corrected *P* < 0.05 were considered as the threshold in seed-to-whole brain analysis. However, in an exploratory approach, ROI-to-ROI gPPI analysis results were reported when uncorrected *P* < 0.05 (two-sided *t*-value > 2).

### Regions of interests and seed definition for generalized psychophysiological interaction analysis

**FIGURE 2:**
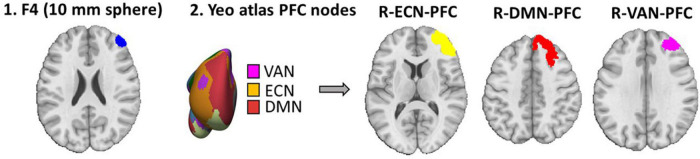
Seed definition for seed-to-whole brain gPPI. Four seeds were defined 1. A 10 mm sphere around the F4 location (center of the anode electrode in EEG 10–20 standard system) in MNI space. 2. Yeo atlas PFC nodes are visualized over an inflated surface. The PFC nodes in ECN, DMN, and VAN that received the highest electric fields were considered seeds for the seed-to-whole brain gPPI analysis.

As shown in Figure 2, for the seed-to-whole brain gPPI analysis, four seeds were defined. Based on atlas-based parcellation of the head models, it was found that PFC nodes in ECN, DMN, and VAN received the highest electric fields (Supplementary Figure 1). The PFC nodes of these three networks in the right hemisphere (near the anode location) were considered seeds. Another seed region was a 10 mm sphere around the F4 site (the center of the anode electrode pad).

In an exploratory approach to determine within and between network changes in task-modulated connectivity, based on Schaefer atlas parcellation (Schaefer et al., 2018), all main nodes of ECN, DMN, and VAN (as depicted and described in the “Atlas-based parcellation of the computational head models” section in Supplementary Material) were considered as a region for ROI-to-ROI gPPI analysis. In Supplementary Figure 1, the main nodes of large-scale networks selected for ROI-to-ROI gPPI are depicted over the brain, and averaged electric fields are reported in each node using bar plots.

### Task-based functional activity analysis

Functional activity was also calculated for all subjects in AFNI using a GLM. The main question was as follows: Is there any relationship between induced electric field (normal or tangential components) in PFC nodes and changes in neural activation (meth > neutral contrast) using fMRI? The correlation between averaged electric fields and changes (post-tDCS minus pre-tDCS) in activation was calculated across the population.

### Self-reported data analysis

Craving for the drug was assessed immediately before and after each MRI scan with the visual analog scale (VAS). Statistical differences between sham and active were calculated to investigate tDCS effects on craving at each time point—changes in craving scores from before to after tDCS were also assessed separately in active and sham conditions. Differences between sham and active were also calculated in terms of PANAS before and after active/sham tDCS.

### Exploratory correlation analysis

The correlation between PPI connectivity and electric fields (both normal and tangential components) within the seed region was calculated for all significant PPI connectivity in an exploratory approach. Furthermore, correlations between PPI connectivity and craving scores were also investigated to determine the association between changes in PPI connectivity and cue-induced craving. These correlations were only focused on the significant PPI connectivity obtained from time by intervention interaction. As an exploratory finding, uncorrected *P*-values are reported for the correlation between neural and behavioral/electric field data.

## Results

All 15 participants reported no adverse effects after the active or sham tDCS. Demographic and substance use profiles at the baseline are presented in Table 1.

**TABLE 1:** Demographic data and substance use profile.

Demographic data (*n* = 15)	Mean ± SD or n (%)
Age (years)	31.33 ± 5.24
Education (years)	11.53 ± 2.40
Duration of meth dependence (years)	3.86 ± 2.22
Duration of meth abstinence (days)	41.06 ± 71.01
Meth use days in the last month before starting abstinence (days)	16.2 ± 10.27
History of cigarette smoking [n (%)]	14 (93.3%)
**Lifetime history of drug abuse before abstinence [n (%)]**	
Opium	14 (93.3%)
Heroin	9 (60.0%)
Crystalline heroin	9 (60.0%)
Alcohol	14 (93.3%)
Cannabis	13 (86.7%)
Cocaine	2 (13.3%)

The sample considered 15 participants with MUD. The values are mean ± standard deviation (SD).

### Computational head modeling simulation results

**FIGURE 3:**
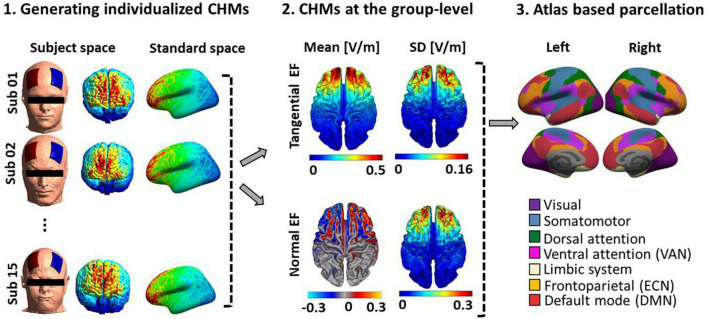
Computational head model analysis. (1) Generating individualized CHMs: Head models were generated for all fifteen participants to calculate electric field distribution patterns using finite element modeling. Individualized CHMs were transformed to standard fsaverage space for group-level analysis. (2) Group-level analysis of CHMs: The mean and standard deviation (SD) of the electric field (for both tangential and normal components) across the population were calculated. (3) Atlas-based parcellation of CHMs (Yeo atlas): Yeo atlas parcellation of CHMs was used to calculate EFs in the main nodes of the three large-scale networks. See Supplementary Figure 1 to depict the Efs in the main nodes of ECN, DMN, and VAN. SD, standard deviation; CHM, computational head model.

As shown in Figure 3, surface-based head models were generated for all fifteen participants, and electric field distribution patterns were calculated based on tangential and normal components. Individualized head models were transformed to fsaverage standard space. Large-scale networks were then extracted from head models based on Yeo atlas parcellation (as shown in Figure 3 panel 3). With respect to the network parcellation of the head models, electric field intensity was calculated in the main nodes of the ECN, DMN, and VAN. Supplementary Figure 1 represents the amount of mean electric field intensity in the main nodes of these networks in each hemisphere.

### Seed-to-whole brain generalized psychophysiological interaction results

**FIGURE 4:**
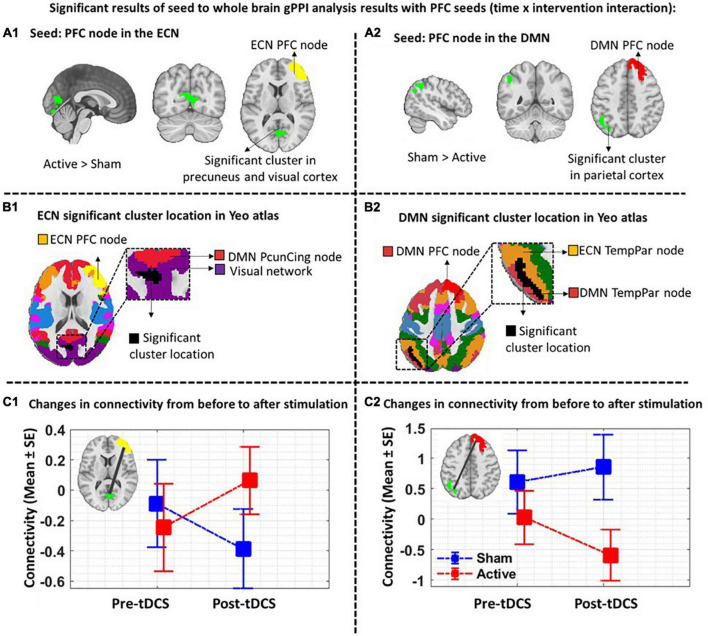
Task-modulated (cue reactivity) connectivity analysis. First row: Significant results of the seed-to-whole brain gPPI analysis results with PFC seeds (time × intervention interaction): The PFC nodes in ECN, DMN, and VAN were used separately for the seed-to-whole brain gPPI analysis. Voxel-level threshold: *P* uncorrected < 0.01 and cluster level threshold: *P* FDR corrected < 0.05. No significant seed to whole-brain gPPI results were found with the VAN PFC node as a seed. Significant clusters with PFC nodes in ECN and DMN are depicted. **(A1)** Significant cluster (located in the visual and the precuneus cortex with 515 voxels and (6, –48, –12) peak coordinate in MNI space) in gPPI analysis with PFC node in ECN. Time × intervention interaction showed increased task-based connectivity in the active tDCS compared to sham **(a2).** Significant cluster (located in parietal cortex with 480 voxels and (–48, –60, 46) peak coordinate in MNI space) in gPPI analysis with PFC node in DMN. Time × intervention interaction showed decreased task-based connectivity in the active condition compared to sham. **(B1,B2).** Significant gPPI clusters with respect to the large-scale network topology in the Yeo atlas. **(C1,C2)**. Connectivity changes from before to after tDCS. Connectivity changes (Mean ± SE) from the first scan (pre-tDCS) to the second scan (post-tDCS) between ECN PFC node and gPPI active cluster (green cluster) (left panel) as well as between DMN PFC node and gPPI active cluster (blue cluster) (right panel) are depicted for both active (in red) and sham (in blue) stimulation conditions. Axial slices are shown in neurological convention. EF, electric field; gPPI, generalized psychophysiological interaction; ECN, frontoparietal network; DMN, default mode network; VAN, ventral attention network. SE, standard error.

As shown in Figure 4, by considering *P* uncorrected < 0.001 at the voxel level and *P* FDR corrected < 0.05 at the cluster level, when the PFC node in the right ECN was used as a seed region, in active > sham contrast, seed-to-whole brain gPPI results showed a significant cluster with 515 voxels in the visual and the precuneus cortex and the coordinate of the cluster’s peak was (6, –48, –12) in MNI space. Our results showed enhanced PPI connectivity between the right PFC node in ECN (as right DLPFC) and the significant cluster (visual cortex and precuneus) after active tDCS, while this connectivity decreased after sham. This task-based connectivity did not show significant differences between sham and active tDCS in pre-stimulation fMRI scans that can be considered as the initial state of the FC (Figure 4 and Table 2). When the PFC node in the right DMN was used as a seed region for seed-to-whole brain gPPI with the same threshold as above, a significant time-dependent interaction was found in the left parietal cortex. The significant cluster that showed decreased PPI connectivity in post-stimulation minus pre-stimulation in active compared to sham tDCS included 480 voxels, and the coordinate for the cluster’s peak was (–48, –60, 46) in MNI space. This task-based PPI connectivity did not show significant differences in the pre-stimulation scan compared to the baseline condition (Figure 4 and Table 2). No significant interaction was found using a 10 mm sphere at the center of the anode or PFC node in VAN in the whole brain analysis.

**TABLE 2:** Significant time by intervention interaction clusters in terms of PPI connectivity obtained from the seed-to-whole brain gPPI analysis.

Seed	Cluster location	Cluster size	Peak coordinate			*t*-value in peak	*P* uncorrected	*P* FDR
			X	Y	Z			
R-ECN-PFC	Right precuneus and visual cortex	515	6	−84	−12	4.59	0.0001	0.015
R-DMN-PFC	Left inferior parietal lobe	480	−48	−60	46	–4.02	0.0003	0.027

*P* uncorrected < 0.01 at the voxel-level and cluster-size *P* FDR corrected < 0.05 at the cluster-level were considered as the threshold. ECN, frontoparietal network; DMN, default mode network.

### Region of interest to regions of interest generalized psychophysiological interaction results

**FIGURE 5:**
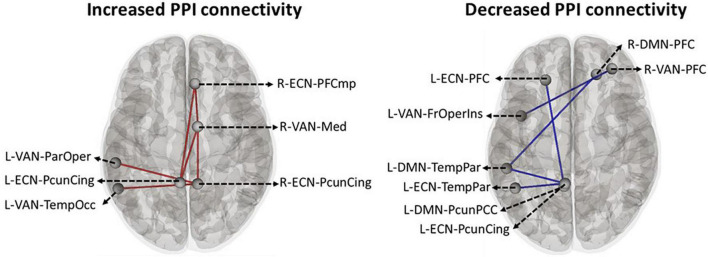
Time by intervention interaction in PPI connectivity within and between large-scale network nodes: Exploratory ROI-to-ROI gPPI results. Task-based modulation in connectivity with time x intervention interaction (*P* uncorrected < 0.05) between the main node of three large-scale networks in the Yeo atlas, including ECN, DMN, and VAN. Within the network, task-based modulation in connectivity did not survive correction using the FDR method. A small dot is an indicator of the main node of a large-scale network. ECN, frontoparietal network; DMN, default mode network; VAN, ventral attention network; FrOperIns, frontal-operculum-insula; Med, medial; ParOper, parietal-operculum; PFC, Prefrontal cortex; TempOcc, tempro-occipital-parietal; TempPar, tempro-parietal; PcunCing, precuneus cingulate; PcunPCC, precuneus posterior cingulate cortex; R, right side; L, left side; blue line, a decreased PPI connectivity between to ROIs in active tDCS while it increased in sham, red line: an enhanced PPI connectivity between two ROIs in active tDCS, while it decreased in the sham.

Based on the main nodes of the ECN, DMN, and VAN (total of 24 ROIs as described in Supplementary Figure 1), ROI-to-ROI gPPI analysis was performed to determine the interaction between time (pre- vs. post-stimulation) and intervention (active vs. sham) in terms of PPI connectivity. Our exploratory results showed significant (P uncorrected < 0.05) changes in PPI within and between large-scale network nodes (Table 3). Main nodes with substantial changes in PPI connectivity are represented with small dots over the gray matter in Figure 5 (the actual topology of each ROI can be found in Supplementary Figure 1). Our results showed enhanced (positive *t*-values) PPI connectivity within ECN nodes and between ECN and VAN nodes after active tDCS compared to sham. However, PPI connectivity within the DMN was decreased (negative *t*-values). We also found decreased PPI connectivity between DMN and VAN nodes in active tDCS compared to sham. Only one PPI connectivity within ECN, from left precuneus-cingulate to left temporoparietal, and one PPI connectivity within VAN, from right PFC to the left fronto-oper-insula, were reduced after active tDCS.

**TABLE 3:** ROI-to-ROI time by intervention interaction in PPI connectivity: Exploratory results.

	Seed name			Target name			*t*-value	*P* uncorrected
	Hemisphere	Net	Node	Hemisphere	Net	Node		
Increased PPI	Right	VAN	Med	Right	ECN	PFCmp	2.64	0.013402
	Left	ECN	PcunCing	Right	ECN	PFCmp	2.55	0.016381
	Right	ECN	PFCmp	Right	VAN	Med	2.48	0.019412
	Left	ECN	PcunCing	Right	VAN	Med	2.42	0.022014
	Right	VAN	Med	Right	ECN	PcunCing	2.39	0.023892
	Right	ECN	PcunCing	Left	VAN	TempOcc	2.3	0.029055
	Right	ECN	PcunCing	Right	VAN	Med	2.22	0.034496
	Left	VAN	ParOper	Right	ECN	PcunCing	2.12	0.043024
Decreased PPI	Right	VAN	PFC	Left	VAN	FrOperIns	–2.46	0.020392
	Left	DMN	TempPar	Left	ECN	PcunCing	–2.45	0.020622
	Left	DMN	PcunPCC	Left	DMN	PFC	–2.41	0.023009
	Left	DMN	TempPar	Right	DMN	PFC	–2.37	0.024863
	Left	DMN	PFC	Left	DMN	PcunPCC	–2.28	0.030705
	Left	ECN	PcunCing	Left	ECN	TempPar	–2.07	0.04799

Main nodes in VAN, ECN, and DMN (a total of 24 ROIs) were considered for an ROI-to-ROI gPPI analysis. Significant time by intervention with *P* uncorrected < 0.05 are reported. Positive *t*-values represent enhanced PPI connectivity changes between seed and target in active compared to sham, while negative *t*-values stand for decreased PPI connectivity changes between seed and target in active compared to sham. ECN, frontoparietal network; DMN, default mode network; VAN, ventral attention network; FrOperIns, frontal-operculum-insula; Med, medial; ParOper, parietal-operculum; PFC, Prefrontal cortex; TempOcc, tempro-occipital-parietal; TempPar, tempro-parietal; PcunCing, precuneus cingulate; PcunPCC, precuneus posterior cingulate cortex; Net, large-scale network (ECN, DMN, or VAN); increased PPI: *t*-value > 0 (an enhanced PPI connectivity between seed and target in active the tDCS, while it decreased in the sham). Decreased PPI: *t*-value < 0 (a decreased PPI connectivity between seed and target in the active tDCS, while it increased in sham).

### Task-based functional activity results

**FIGURE 6:**
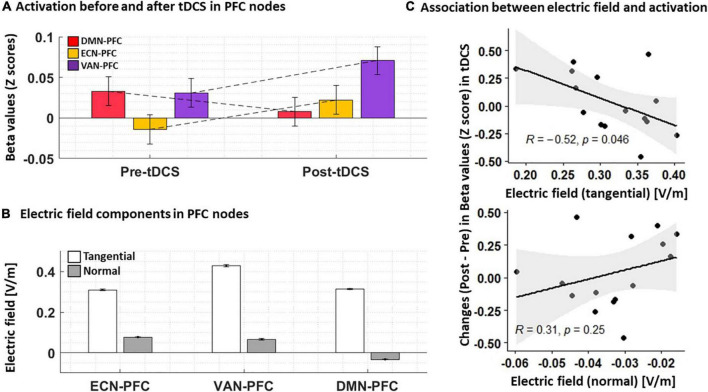
**(A)** Functional activation before and after tDCS. Normalized beta values (Z score) obtained from first-level general linear models for each individual in PFC nodes are visualized pre- and post-tDCS. **(B)** Electric field components. Tangential and normal components of the electric fields in PFC nodes are extracted for each individual. **(C)** Association between changes in neural activation and electric fields. A Scatter plot for the PFC node in DMN showed a significant correlation between changes in neural activation and the tangential component of the electric field. Bars show average values, and error bars indicate standard errors.

Changes in functional activity were also checked in PFC nodes. Our results (Figure 6) showed that functional activity in the right PFC node in DMN decreased after tDCS while activity in the PFC nodes of ECN and VAN increased (all changes were insignificant). Furthermore, we found a negative averaged electric field in DMN while the direction of the averaged electric field was inward for both ECN and VAN. The tangential electric field also showed a negative significant correlation with changes in neural activation (*r* = –0.52, *P* = 0.046).

### Self-reported results

**FIGURE 7:**
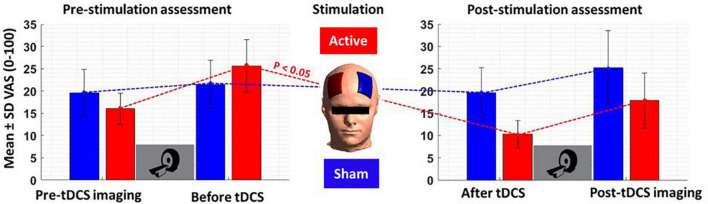
Self-reported craving. Self-reported craving based on visual analog scale [VAS (0–100)] in four main time points; pre-tDCS imaging (before the first scan), before tDCS (after the first scan), after tDCS (before the second scan), and post-tDCS imaging (after the second scan). Bars show mean values, and error bars show the craving score’s standard deviation (SD). The craving score was significantly (*P* < 0.05) decreased from before to after tDCS only in the active tDCS. The blue color is related to the sham condition, and the red is related to active tDCS.

Subjective craving scores showed a significant reduction in immediate craving after active [mean ± standard error (SE) = –15.42 ± 5.42] compared to sham (mean ± SE = –1 ± 2.63) stimulation (Figure 7). To ensure that results did not correspond to mood changes, PANAS were compared between sham and active conditions at two different time points; before and after tDCS. No significant differences (*P* > 0.05) were found between sham and active stimulation. More details on self-reported data can be found in Table 4.

**TABLE 4:** Self-report data.

Self-report variable	Assessment time	tDCS (Mean ± SD)		*P*-value
		Active	Sham	
Self-report of craving VAS (0–100)	Pre-tDCS-imaging	16.02 ± 0.89	19.56 ± 1.35	0.64
	Before tDCS	25.58 ± 1.52	21.01 ± 1.39	0.39
	After tDCS	10.16 ± 0.77	19.92 ± 1.45	0.10
	Post-tDCS imaging	17.87 ± 1.6	25.20 ± 2.15	0.15
Positive affect score (PANAS)	Before tDCS	34.73 ± 0.52	32.60 ± 0.39	0.09
	After tDCS	33.93 ± 0.58	34.13 ± 0.66	0.75
Negative affect score (PAVAS)	Before tDCS	25.60 ± 0.59	25.86 ± 0.69	0.97
	After tDCS	25.13 ± 0.63	26.80 ± 0.66	0.70

Differences between active and sham tDCS in terms of VAS and PANAS. Un-corrected *P*-values are reported based on the Wilcoxon test to compare active and sham conditions at each time point. VAS, visual analog scale; PANAS, positive and negative affect scores.

### Correlation results

In an exploratory approach, correlation analysis was performed to find if there was any significant (*P* uncorrected < 0.05) association between neural response (PPI connectivity during cue exposure) and self-reported craving (active vs. sham) or tDCS-induced electric fields (in active tDCS). The PPI connectivity with significant time by intervention interaction obtained from the ROI-to-ROI gPPI analysis showed significant relationships with changes in craving scores. PPI connectivity changes (post minus pre) between precuneus-cingulate node in right ECN and medial node in right VAN (Figure 5 and Table 3) showed a negative significant correlation with changes in VAS after active tDCS (active: *R* = –0.75, *P* uncorrected = 0.005, sham: *R* = –0.091, *P* uncorrected = 0.78). This negative correlation means higher PPI connectivity was associated with less induced craving. Furthermore, changes in PPI connectivity between the temporoparietal node in the left DMN and the precuneus-cingulate node in left ECN (Figure 5 and Table 3) also showed a significant positive correlation with changes in VAS after active tDCS (active: *R* = 0.6, *P* uncorrected = 0.04, sham: *R* = 0.033, *P* uncorrected = 0.92). This positive correlation means that less connectivity was associated with less induced craving. A trend toward significant correlation was found between the normal component of the electric field in the precuneus-cingulate node in the right ECN and changes in PPI connectivity between this node and the temporal-occipital node in the left VAN (Figure 5 and Table 3) (*R* = –0.51, *P* uncorrected = 0.053).

## Discussion

This investigation examining the effects of bilateral DLPFC stimulation with tDCS in a group of participants with MUDs yielded four main results. First, in time by intervention interaction analysis of the seed-to-whole brain gPPI, the PFC node in ECN showed a significant enhancement in PPI connectivity with visual, and the precuneus cortex [(6, –84, –12) with 515 voxels] and the PFC node in DMN showed a decreased PPI connectivity with the parietal cortex (–48, –60, 46) with 480 voxels); voxel-level threshold *P* uncorrected < 0.001, and cluster-level threshold: *P* FDR corrected < 0.05. Second, our exploratory (*P* uncorrected < 0.05) findings showed increased PPI connectivity within and between ECN and VAN, while connectivity between ECN and DMN and within DMN was decreased. Third, DLPFC stimulation with anode/cathode over F4/F3 location reduced cue-induced craving significantly after active tDCS (*P* < 0.05), while sham tDCS did not change the craving score significantly. Fourth, ECN-VAN PPI connectivity showed a significant (*P* uncorrected < 0.05) negative correlation with craving (higher connectivity induced lower craving). In comparison, the correlation was negative between craving and ECN-DMN connectivity (higher connectivity induced higher craving). Finally, we found a correlation between the normal component of the electric field and changes in neural and behavioral responses. Taken together, these results indicate that the effects of tDCS do not appear to be limited to the stimulation site, and network-level connectivity and electric field distribution patterns at the network level should be considered in task-modulated neurostimulation studies.

In line with previous findings in the field of tDCS-SUDs, we found a significant reduction in self-reported craving after active tDCS without any significant change after sham stimulation (Figure 6). Despite heterogeneity in the effects of brain stimulation on drug craving, a previous meta-analysis reported positive potentials for tDCS effects on cue-induced craving (Jansen et al., 2013; Song et al., 2019; Kim and Kang, 2020). A recent systematic review and meta-analysis revealed positive tDCS effects on alcohol craving, specifically in bilateral DLPFC protocols with the anode/cathode over the right/left DLPFC (similar to the montage used in this study) (Kim and Kang, 2020). Furthermore, the effectiveness of tDCS in reducing drug cravings was reported in several previous studies related to SUDs, including methamphetamine (Shahbabaie et al., 2014), nicotine (Fregni et al., 2008; Hajloo et al., 2019), alcohol (Boggio et al., 2008; Klauss et al., 2018), and marijuana (Boggio et al., 2010) use disorders. However, despite the positive effects of tDCS on drug craving/consumption, some previous findings reported no statistically significant reduction of craving after active tDCS compared to sham (Xu et al., 2013; Lupi et al., 2017; Mondino et al., 2018; Claus et al., 2019). Compared to our stimulation protocol, these studies used different electrode sizes and positions for targeting DLPFC, such as squared shape 11 cm^2^ electrodes over F10 as anode and contralateral upper arm as a cathode (Claus et al., 2019), or a 35 cm^2^ anode midway between F4 and Fp2 and a 100 cm^2^ cathode over the left occipital region in midway between O1 and T5 (Mondino et al., 2018), or 35 cm^2^ anode/cathode over F3/Fp2 (Xu et al., 2013) in EEG standard system. Electrode montage (including electrode position, size, and orientation) can affect electric field distribution patterns, and our results [in line with previous dose-response findings (Kim et al., 2014; Kasten et al., 2019)] suggest that the electric field distribution patterns are a relevant factor for modulating brain activity and corresponding physiological and behavioral effects like craving. Other methodological aspects (e.g., number of participants, number of active sessions, and duration of each session), drug dependency profiles (e.g., type of substance, duration of abstinence, and state of dependency), and brain structural alterations in regions associated with SUDs [e.g., different cortical morphology (McCalley and Hanlon, 2021; Soleimani et al., 2021b)] may also affect stimulation outcomes in the field of addiction.

Dysfunction in the prefrontal cortex has been linked to loss of control over drug consumption and craving as a critical step in the progression of SUD (George and Koob, 2010). Our atlas-based parcellation of head models supports the finding that in a bilateral DLPFC stimulation with large electrode pads, PFC nodes in three large-scale networks, including ECN, DMN, and VAN, are highly stimulated (Supplementary Figure 1), which can modulate other parts of the brain through within/between functional network coupling and a complicated mosaic of interactions between network nodes. Distribution of the electric fields and FC alterations at the network level are consistent with previous studies that reported brain regions do not respond to brain stimulation in isolation, and many distributed areas interact with each other through the brain networks (Keeser et al., 2011; Reithler et al., 2011). More focal electrode montages, such as high-definition (e.g., cross-like 4 × 1 montage with four peripheral and one central electrode of opposing polarity) (Datta et al., 2009) or multi-array electrodes (Fischer et al., 2017), could potentially provide the possibility for more refined control of the network-level modulation with tDCS interventions. Although all three networks received a high level of electric field intensity in the PFC nodes, our results showed that the PFC node in DMN, in contrast to the PFC nodes in ECN and VAN, received an electric field with an outward direction (negative normal component). Different directions of the electric fields between DMN and ECN/VAN networks may affect network-based responses to tDCS.

Previous findings reported the different associations between large-scale brain network activity/connectivity and factors related to SUDs (Zhang and Volkow, 2019). For example, in the PPI analysis of the frontoparietal network, in participants with alcohol use disorder, weaker FC between the striatum, anterior insula, and prefrontal cortex was related to greater alcohol use disorder severity (Courtney et al., 2013). Our findings also showed that tDCS could modulate drug-related connectivity at the network level. Increased FC between the PFC node in ECN and precuneus and visual cortex was found in our seed-to-whole brain analysis. The precuneus has connectivity with different networks, including DMN, ECN, visual and motor cortex (White et al., 2010; Allen et al., 2011), and increased PPI connectivity with the precuneus cortex after active tDCS can be considered as an intermediate node that modulates communication between other network nodes through excitatory/inhibitory pathways that can be modulated by tDCS.

Indeed, when the PFC node in the right DMN was used as a seed, we observed a decreased inter-hemispheric frontoparietal connectivity in active tDCS compared to sham. tDCS-induced inter-hemispheric change in task-based connectivity is an important finding. It largely depends on the contribution of the cathode electrode, which is located in the left hemisphere (Sehm et al., 2012, 2013). In this study, PPI connectivity was decreased predominantly between the DMN-PFC node in the right hemisphere and the temporoparietal nodes of the left DMN and ECN after active tDCS. This diminished connectivity within DMN in response to tDCS, while people are exposed to drug cues, is consistent with previous tDCS studies in SUDs that reported decreased resting-state connectivity within DMN after active tDCS compared to sham (Shahbabaie et al., 2018a). Although DMN exhibits higher activity at rest than during the task, growing evidence shows DMN is also involved in goal-directed tasks when self-related cognition (e.g., self-referential judgment) is needed (Buckner et al., 2008; Andrews-Hanna et al., 2010). Previous studies have demonstrated that functional activity and connectivity within the DMN are reduced during executive functions. This reduction is associated with increased activation in the task-positive region such as ECN (Fox et al., 2005). Alteration coupling between DMN and ECN during cue exposure may be induced by the mediation effect of the VAN salience network based on changing resource allocation between ECN and DMN (Zhang et al., 2017), and our ROI-to-ROI results corroborate this assumption. However, no seed-to-whole brain PPI changes with the PFC node in VAN passed the statistical threshold. Smaller PFC nodes in VAN compared to PFC nodes in ECN or DMN may have contributed to the lack of significant change within VAN.

Our exploratory ROI-to-ROI results that did not survive multiple comparisons correction suggest the potential for tDCS in the alteration of task-based connectivity within and between ECN, DMN, and VAN that play a vital role in cognitive functions in SUDs (Weiland et al., 2014; Geng et al., 2017; Yang et al., 2017). In a previous study on abstinent smokers compared with subjects who relapsed, PPI analysis showed that PPI connectivity during a cue-reactivity task increased between the anterior cingulate cortex (as a main node in VAN) and DLPFC (as a main node in ECN) in abstinent smokers (Janes et al., 2010). The enhanced PPI connectivity within ECN and between ECN and VAN (both within and between hemispheric) (Figure 5 and Table 3) may suggest elevated information processing within these networks and increased engagement of ECN to mediate cognitive control process induced by more allocation of attentional resources (increased connectivity between VAN and ECN) after active tDCS (Liang et al., 2016). On the contrary, we dominantly found decreased PPI connectivity in the main nodes of the DMN in the left hemisphere in active compared to sham stimulation, which can be attributed to two main reasons: (1) the effectiveness of cathodal stimulation over the left DLPFC and (2) the role of the DMN as a task-negative network and its counterbalance interaction with ECN activity/connectivity (Wang et al., 2019).

Significant correlations between PPI connectivity in central nodes of large-scale networks and craving scores suggest that tDCS might be reinforcing the coupling/decoupling of ECN, DMN, and VAN during the accomplishment of cue exposure tasks, which is then associated with a lower craving score. However, our results, in line with previous findings in the field of NIBS protocols, suggest inter-individual variability in response to tDCS. For example, López-Alonso et al. (2014) observed that only 45% of subjects responded to anodal tDCS as expected when the motor cortex was targeted. One of the main sources of variation is electric field distribution patterns. Our results suggest that the normal component of the electric fields may also have a critical role in the neural response to tDCS. However, a larger sample size is needed to confirm the correlation between neural/behavioral responses and different components of tDCS-induced electric fields. Although we found that some could not find a trivial relationship between tDCS-induced electric fields, self-reported data, and neural response at the network level, there may be other brain regions and psychological functions for which the relationship between connectivity, behavior, and electric field distribution is divergent in SUDs—more comprehensive research is needed to investigate these associations.

### Limitations and future works

This study should be interpreted in light of potential limitations. First, the sample size is relatively small, and additional investigation involving a larger sample size and more diverse participants (e.g., including females to study the potential role of sex differences and other types of substance use) is needed to generalize the results of this study. However, despite the small sample size, a cross-over design used in this study enhanced the power of analysis by comparing equivalent subjects in each group (Cleophas and de Vogel, 1998). Second, in this study, we focused on the ECN, DMN, and VAN as the large-scale core networks in SUDs, and we did not explore whether other networks and neural circuits are modulated during a cue-reactivity task after tDCS. Furthermore, we only used PFC nodes in these networks (located near the anode) for the seed to whole-brain analysis based on maximum averaged electric fields obtained from head models. However, other predefined ROIs that play essential roles in drug cue reactivity (e.g., subcortical regions such as the insula or ventral striatum) may be modulated indirectly (e.g., through top-down regulation) by tDCS and can be explored in future task-modulated studies. Third, this study used conventional large electrode pads (5 × 7 cm^2^) for bilateral DLPFC stimulation. Because of the diffusivity of the current in conventional tDCS, more complicated interactions might be induced between and within large-scale networks. The focal stimulation of the network nodes using conventional electrodes is complex. More focal electrode arrangements (high definition or multi-array electrodes) might be a better candidate for future tDCS studies. Fourth, this study used a one-size-fits-all electrode arrangement based on anatomical targeting that increases inter-individual variability in response to tDCS. In future studies, customized electrode arrangements, such as fMRI-guided multi-electrode montages, can be used for each individual to target specific brain functions related to cue-induced craving, as suggested in previous NIBS research (Hanlon et al., 2018; Cash et al., 2020). Fifth, here, we only used T1-weighted images for generating computational head models since other types of anatomical images were not available in our database. Considering T2-weighted images (Van Hoornweder et al., 2022), computed tomography (CT) scans (Puonti et al., 2020) or diffusion tensor images (Suh et al., 2012) may increase the accuracy of the segmentation, mesh generation, and electric field calculations. However, computational head models generated by T1-weighted images were validated with in-vivo measurements (Huang et al., 2017), and numerous published studies, even in dose-response relationship analysis, performed electric field simulations based solely on T1-weighted images (Boayue et al., 2018; Muffel et al., 2019; Suen et al., 2021). Finally, considering more advanced data acquisition [e.g., using multi-session tDCS trials or collecting fMRI data during the application of tDCS (concurrent tDCS-fMRI)], more advanced analysis methods rather than linear analysis (e.g., non-linear analysis method instead of correlation or regression analysis in this study), and integrating the results with other neuroimaging modalities (e.g., resting-state data to find a correlation between resting-state and task-based connectivity or diffusion tensor imaging for creating more precise computational head models) might also be beneficial in future studies.

## Conclusion

Taken together, the current study provides new insight into the neural circuitry underlying MUDs that can be modulated by active tDCS during a drug cue exposure task. Alterations in ECN-DMN-VAN functional coupling may be critical in behavioral alterations that underlie drug dependence. As the most remarkable contribution of this paper, we have suggested that network-level PPI connectivity during a cue-reactivity task can be applied as a predictive biomarker for investigating responsiveness to tDCS. We also suggested that brain function alterations in response to tDCS may be related to the direction of the electric field. This highlights the importance of personalized computational head modeling methods in tDCS studies. However, test-retest studies are needed to demonstrate its reproducibility and clinical significance. Given the relevance of functional activity/connectivity of the brain networks in psychological, physiological, and pathological states, this approach may offer new possibilities for discovering stimulation targets in healthy and patient populations. Network-based connectivity patterns could inform electrode placement in future studies that may help initiate image-guided personalized brain stimulation by considering each individual’s brain structure and brain function.

## Data availability statement

The raw data supporting the conclusions of the article are available on request to the corresponding authors.

## Ethics statement

The studies involving human participants were reviewed and approved by the Ethics Committee of the Tehran University of Medical science and all subjects gave their written consent to the experiment according to the Declaration of Helsinki. The trial was registered at the WHO registry for clinical trials (IRCT code: 2012102011172N1). The patients/participants provided their written informed consent to participate in this study.

## Author contributions

GS and HE designed the study. GS performed the simulations and data analysis under HE and FT supervision. GS wrote the manuscript with input from HE, FT, and MO. All authors contributed to manuscript preparation and agreed to the final manuscript before submission.

## Footnotes

1
http://surfer.nmr.mgh.harvard.edu

2
https://www.fil.ion.ucl.ac.uk/spm/
