# Supplementary material for: DLPFC stimulation alters large-scale brain networks connectivity during a drug cue reactivity task: A tDCS-fMRI study
Source: Front Syst Neurosci. 2022 Oct 6;16:956315. doi: 10.3389/fnsys.2022.956315 (PMC9582757; doi:10.3389/fnsys.2022.956315)
Supplement: Supplementary file 1 [file Data_Sheet_1.docx]

Supplementary Material

# Supplementary Data

## Atlas-based parcellation

head models and functional maps were parcellated based on Yeo atlas. In the next step, Schaeffer-400-2018 was applied for a finer parcellation of the networks and subregions that were placed adjacent to each other were merged to form the main nodes of the networks. In this way, ECN included five main nodes (L stands for the left hemisphere): orbitofrontal cortex (L-ECN-OFC), Precuneus-cingulate (L-ECN-PcunCing), posterior-medial prefrontal cortex (L-ECN-PFCmp), and tempro-parietal (L-ECN-TempPar). posPFCmp R-ECN-TempPar. The DMN included three main nodes in each hemisphere: precuneus-posterior cingulate cortex (L/R-DMN-PcunPCC), L/R-DMN-PFC, and L/R-DMN-TempPar. Left VAN was divided into five main nodes: frontal-operculum-insula (L-VAN-FrOperIns), medial (L-VAN-Med), parietal-operculum (L-VAN-ParOper), prefrontal cortex (L-VAN-PFC), and tempro-occipital (TempOcc). Right VAN was also parcellated into four main subregions (R stands for the right hemisphere): R-VAN-FrOperIns, R-VAN-Med, R-VAN-PFC, and tempro-parietal-occipital (TempOccPar).

# Supplementary Figures and Tables

## Supplementary Figures

**
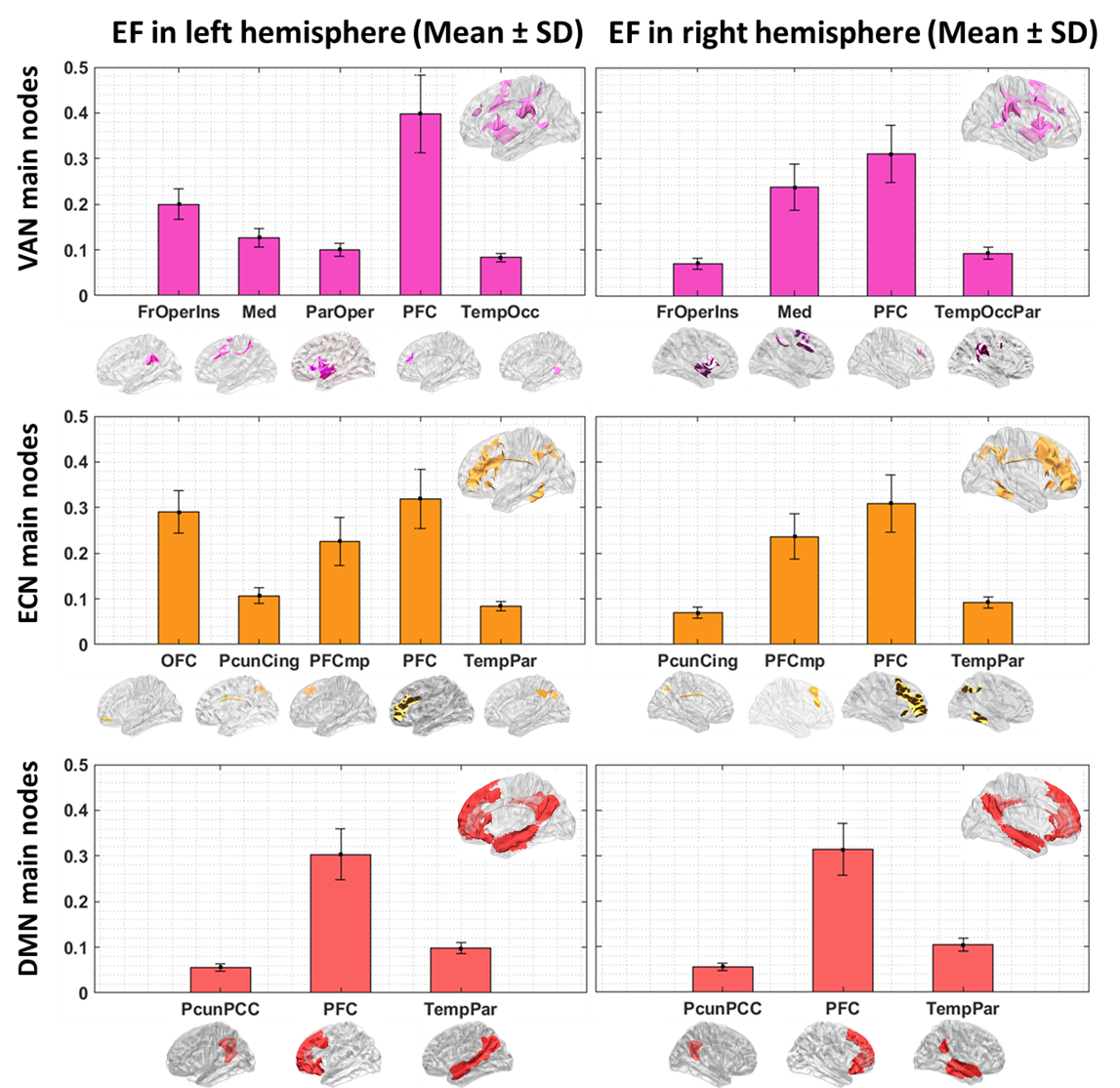
**

**Figure S1:** electric field intensity in main nodes of three large-scale brain networks (VAN, ECN, and DMN) at a current of 2 mA generate by each montage. Bars show mean values and error bars represent SDs of the electric field intensity in volt per meter ([V/m]) across 15 participants inside the main nodes of large-scale networks for F4-F3 electrode montages in the left and right hemisphere of each network. Three main networks in Yeo atlas including ventral attention network (First row; VAN), frontoparietal network (second row; ECN), and default mode network (third row, DMN) parcellated into subregions based on Schaefer2018_400Parcells_7networks atlas and main nodes of each network were extracted by averaging the EFs in the subregions of the main nodes. Labels below the horizontal axis denote the name of main nodes inside the large-scale network and small brains next to the labels represent the spatial location of the main nodes in fsaverage space. Abbreviations: EF: electric field; SD: standard deviation. VentAtn: ventral attention network; ECN: frontoparietal network; DMN: default mode network. FrOperIns: frontal-operculum-insula, Med: medial, ParOper: parietal-operculum, PFC: Prefrontal cortex, TempOcc: tempro-occipital-parietal, OFC: orbitofrontal cortex, TempPar: tempro-parietal PcunCing: precuneus cingulate, PcunPCC: precuneus posterior cingulate cortex
